# ABCs of Insect Resistance to Bt

**DOI:** 10.1371/journal.pgen.1005646

**Published:** 2015-11-19

**Authors:** Bruce E. Tabashnik

**Affiliations:** Department of Entomology, University of Arizona, Tucson, Arizona, United States of America; Fred Hutchinson Cancer Research Center, United States of America

Genetically engineered crops represent one of the most controversial and rapidly adopted technologies in the history of agriculture. To improve pest control, scientists have engineered cotton, corn, and soybeans to make insecticidal proteins from the common bacterium *Bacillus thuringiensis* (Bt) [1]. These Bt toxins kill some devastating pests, but unlike broad-spectrum insecticides, they do little or no harm to most other organisms, including people [2,3]. The original Bt crops, first commercialized in 1996, each made a single crystalline (Cry) toxin from the Cry1 family effective against certain lepidopteran larvae. However, some of the environmental, health, and economic benefits of Bt crops have been lost because of rapid evolution of pest resistance, particularly to single-toxin Bt crops (Fig 1) [4].

**Fig 1:**
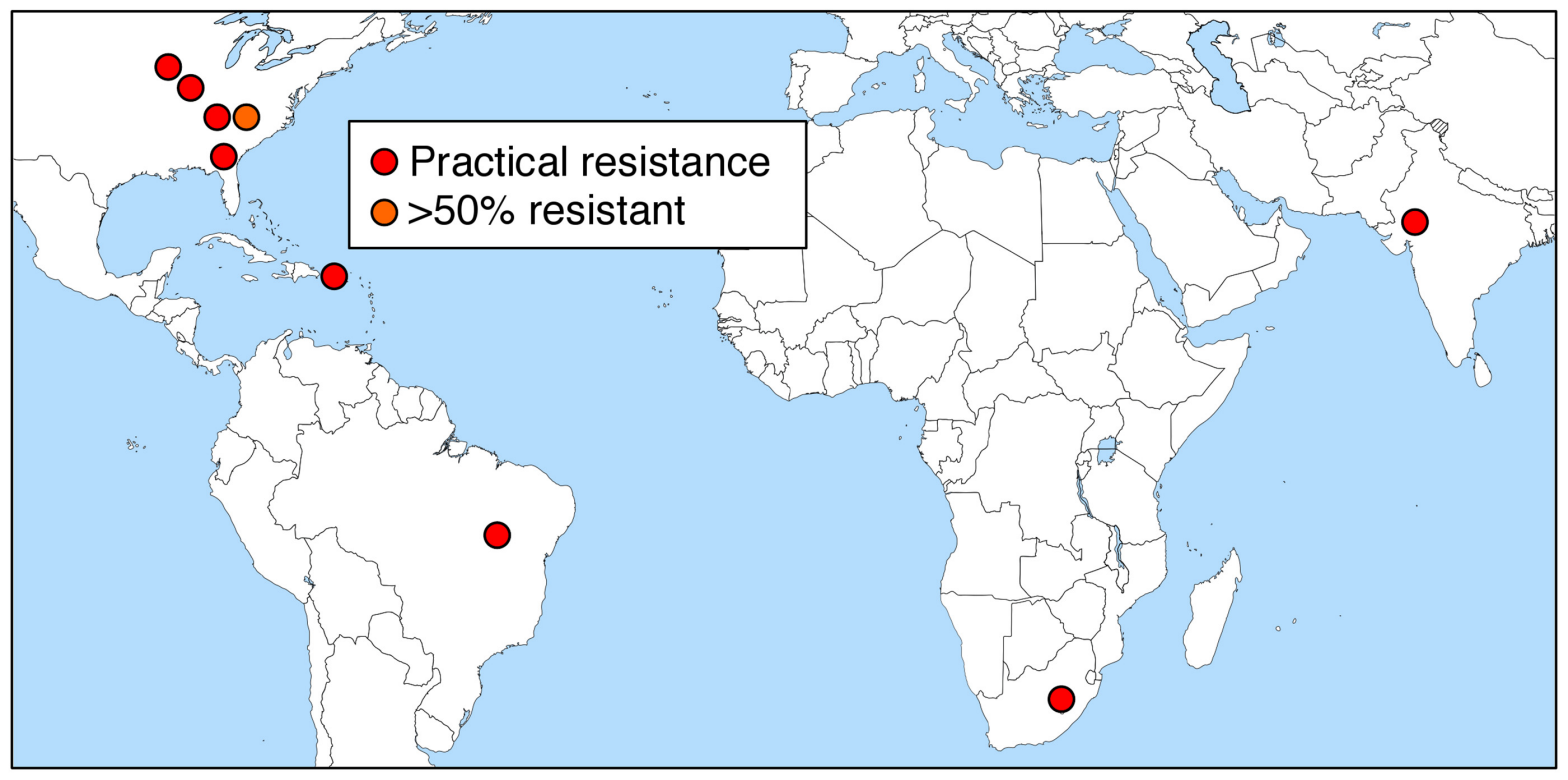
Field-evolved resistance to Bt crops [4,20,27,29–31]. The red circles indicate practical resistance, where scientists have reported one or more field populations with >50% resistant individuals and reduced efficacy of the Bt crop that has practical consequences for pest control. Six of these eight cases entail insects resistant to Cry1 toxins: four against Bt corn (*Spodoptera frugiperda* resistance to Cry1F in Puerto Rico, Brazil, and the continental United States and *Busseola fusca* resistance to Cry1Ab in South Africa) and two against Bt cotton (resistance to Cry1Ac of *Helicoverpa zea* in the US and *Pectinophora gossypiella* in India). The other two cases of practical resistance are *Diabrotica virgifera virgifera* resistance to Cry3Bb and mCry3A in the midwestern US. The orange circle indicates >50% resistant individuals with reduced efficacy expected (but not reported) for *H*. *zea* resistance to Cry2Ab in multi-toxin Bt cotton in the US. The number of cases with >50% resistant individuals (red or orange) increased from one in 2005 to nine in 2013—the most recent year for which monitoring data are generally available.

To delay resistance and broaden the spectrum of pests controlled, newer “second generation” Bt crops produce two or more Bt toxins [5]. In particular, Bt toxin Cry2Ab from the Cry2 family is used widely in combination with Cry1 toxins to kill caterpillar pests. For example, the percentage of all cotton planted that was Bt cotton producing both Cry1Ac and Cry2Ab was 69% in the US in 2012, 91% in India in 2013, and 94% in Australia in 2011 [6–8]. Despite the use of Cry2Ab in multi-toxin Bt crops since 2003 and in multi-toxin Bt sprays for decades, nearly all of what we know about Bt toxins is based on the Cry1 family. In a breakthrough that promises to accelerate progress in understanding Cry2 toxins, Tay et al., in this issue of *PLOS Genetics* [9], identify a gene tightly linked with resistance to Cry2Ab in *Helicoverpa armigera*, one of the world’s most damaging crop pests.

The advance by Tay et al. is the fruit of more than a dozen years of a synergistic collaboration, integrating results from classical and molecular genetics. As part of Australia’s proactive program for monitoring resistance to Bt crops, screening of field populations for resistance to Cry2Ab began in 2002, two years before farmers there started planting Bt cotton producing this toxin in combination with Cry1Ac. Using a method called the F_2_ screen, the second generation progeny of single pairs of field-collected insects were tested on an artificial diet treated with Cry2Ab. In the first year of screening, the Australian team detected resistance to Cry2Ab in one of the 28 isofemale lines that were tested [10]. The 17 survivors of exposure to Cry2Ab from this isofemale line became the progenitors of a Cry2Ab-resistant strain (SP15) that was repeatedly crossed with a susceptible strain and selected with Cry2Ab [11]. The SP15 strain was so resistant that it suffered little mortality when exposed to the highest concentration of Cry2Ab tested in the artificial diet [11]. Bioassays of progeny from crosses indicated this resistance to Cry2Ab was autosomal, recessive, and probably conferred by a single genetic locus [11].

Tay et al. used genetic linkage analysis with molecular markers to narrow the source of resistance to Cry2Ab in this strain to a chromosomal region containing less than 30 genes. They found that two of these genes encode the ATP-binding cassette (ABC) transporter proteins ABCA1 and ABCA2. These genes were prime suspects because resistance to Cry1 toxins is linked with the ABC transporter protein ABCC2 in strains of seven species of Lepidoptera, including *H*. *armigera* [12–15].

Tay et al. honed in on ABCA2 because it was produced in the midgut where Bt toxin binding occurs, but ABCA1 was not. In cDNA of ABCA2 in a resistant individual from the SP15 strain, they found a 73 base pair deletion that introduces a premature stop codon. Including the SP15 strain, Tay et al. detected the same mutation in five of seven resistant lines of *H*. *armigera*, each established independently from insects collected from the field during 2002 to 2012. They found two other mutant alleles at the same locus in two other resistant lines, yielding a total of three mutant alleles that each encode a truncated ABCA2 protein. Screening of one Cry2Ab-resistant strain of the congeneric species *Helicoverpa punctigera* revealed a different premature stop codon in the orthologous gene.

This new paper is a worthy successor to the landmark 2001 article by David Heckel’s group, which was the first to report the molecular genetic basis of resistance to Cry1Ac [16]. As that paper did for Cry1A toxins, this one will accelerate research to enhance understanding of the mode of action of Cry2A toxins. We now know that diverse mutations in cadherin and other genes can confer resistance to Cry1Ac in the field [17,18], which limits the utility of PCR-based monitoring for specific resistance mutations. Nonetheless, identification of cadherin as a key receptor for Cry1A toxins did spur genetic engineering of modified toxins that kill some insects resistant to Cry1 toxins [19,20], and this paper might inspire analogous discoveries for Cry2-resistant insects.

Despite the ≥85% adoption of Bt cotton producing Cry2Ab and Cry1Ac in Australia since 2005 [7], eight years of monitoring data from the robust F_1_ screen method show no significant increase in the frequency of resistance to Cry2Ab (0.032 in 2007–2008 to 0.021 in 2014–2015 for *H*. *armigera*; 0.010 to 0.011 over the same eight years for *H*. *punctigera*) [21]. If the Cry2Ab resistance alleles provide protection against this toxin, why has their frequency not increased? Part of the explanation is that Cry1Ac resistance remains rare in Australia [21] and, with little or no cross-resistance, the Cry1Ac in the two-toxin cotton kills individuals resistant to Cry2Ab. This is reflected in only zero to 8.5% survival of SP15 larvae on two-toxin cotton [22].

Fitness costs associated with Cry2Ab resistance alleles could also delay the evolution of resistance in *H*. *armigera* and *H*. *punctigera* by selecting against these alleles when larvae eat non-Bt cotton or any of the other non-Bt host plants of these polyphagous species. Because insect ABC transporters often provide protection against xenobiotics, the resistance-conferring mutations disrupting these proteins may diminish their natural function, yielding higher fitness costs in the presence of toxic substances, such as plant defensive compounds and insecticides other than Bt toxins [12]. Although significant fitness costs of Cry2Ab resistance in *H*. *armigera* were not detected when larvae ate either artificial diet [23] or mature non-Bt cotton [22], survival on younger non-Bt cotton was significantly lower for resistant larvae (81%) than susceptible larvae (100%) [22], which is a substantial fitness cost. Moreover, in strains of *H*. *armigera* and *H*. *punctigera* with ABCA2 mutations, resistance to Cry2Ab is associated with significantly increased susceptibility to the organophospate insecticide chlorpyrifos and the carbamate insecticide methomyl [24].

Although Tay et al. provide compelling evidence that ABCA2 is essential for toxicity of Cry2Ab to *Helicoverpa armigera*, the precise role of this protein in the mode of action of Cry2Ab remains unknown. In strains of *H*. *armigera* and *H*. *punctigera* that harbor ABCA2 mutations, resistance to Cry2Ab is associated with reduced binding of this toxin [25], which implies that ABCA2 either binds Cry2Ab directly or facilitates binding of Cry2Ab to other target sites. A similar correlation with reduced toxin binding is typically seen with resistance to Cry1A toxins linked with mutations disrupting ABCC2 [12]. However, in the silkworm *Bombyx mori*, Cry1Ab bound equally to brush border membrane vesicles from susceptible larvae and larvae with ABCC2-linked resistance [26]. Additional work is needed to test the hypotheses of Tay et al. that ABCA2 provides both binding and pore formation functions for Cry2Ab.

It will also be important to determine if resistance to Cry2Ab is associated with mutations affecting ABCA2 in other lepidopteran pests, particularly the field-evolved resistance to Cry2Ab in the US of *Helicoverpa zea* [4], a close relative of *H*. *armigera*. With global use of Cry2Ab increasing, more cases of field-evolved resistance are inevitable. For example, the risk is high for resistance of pink bollworm (*Pectinophora gossypiella*) to Cry2Ab in India, where the refuges of non-Bt host plants are scarce, resistance to Cry1Ac is widespread, and exposure to Cry2Ab is extensive [8,18,27,28]. Better understanding of the role of ABCA2 in the mode of action and mechanism of resistance to Cry2Ab may enhance our capacity to counter such resistance.

## References

[1] James C. Global status of commercialized biotech/GM crops: 2014. ISAAA Brief No. 49. ISAAA: Ithaca, NY. 2014.

[2] Sanahuja G, Banakar R, Twyman R, Capell T, Christou P. *Bacillus thuringiensis*: A century of research, development and commercial applications. Plant Biotechnol J. 2011; 9:283–300. 10.1111/j.1467-7652.2011.00595.x 21375687

[3] Comas C, Lumbierres B, Pons X, Albajes R. No effects of *Bacillus thuringiensis* maize on nontarget organisms in the field in southern Europe: a meta-analysis of 26 arthropod taxa. Transgenic Res. 2014; 23:135–143. 10.1007/s11248-013-9737-0 23904218

[4] Tabashnik BE, Brévault T, Carrière Y. Insect resistance to Bt crops: lessons from the first billion acres. Nat Biotechnol. 2013; 31: 510–521. 10.1038/nbt.2597 23752438

[5] Carrière Y, Crickmore N, Tabashnik BE. Optimizing pyramided transgenic Bt crops for sustainable pest management. Nat Biotechnol. 2015; 33:161–168. 10.1038/nbt.3099 25599179

[6] Brévault T, Heuberger S, Zhang M, Ellers-Kirk C, Ni X, Masson L., et al Potential shortfall of pyramided transgenic cotton for insect resistance management. Proc Natl Acad Sci USA 2013; 110: 5806–5811. 10.1073/pnas.1216719110 23530245 PMC3625267

[7] Downes S, Mahon R. Evolution, ecology and management of resistance in *Helicoverpa* spp. to Bt cotton in Australia. J Invert Pathol. 2012; 110:281–286. 10.1016/j.jip.2012.04.005 22537836

[8] Choudhary B, Gaur K. Biotech cotton in India, 2002 to 2014: adoption, impact, progress & future ISAAA: Ithaca, NY 2015.

[9] Tay WT, Mahon RJ, Heckel DG, Walsh TK, Downes S, et al Insect resistance to *Bacillus thuringiensis* toxin Cry2Ab is conferred by mutations in an ABC transporter subfamily A protein. PLoS Genet. 2015; 11(11): e1005534 10.1371/journal.pgen.1005534 26583651 PMC4652872

[10] Mahon RJ, Olsen KM, Downes S, Addison S. Frequency of alleles conferring resistance to the Bt toxins Cry1Ac and Cry2Ab in Australian populations of *Helicoverpa armigera* (Lepidoptera: Noctuidae). J Econ Entomol. 2007; 100: 1844–1853. 18232402 10.1603/0022-0493(2007)100[1844:foacrt]2.0.co;2

[11] Mahon RJ, Olsen KM, Garsia KA, Young SR. Resistance to *Bacillus thuringiensis* toxin Cry2Ab in a strain of *Helicoverpa armigera* (Lepidoptera: Noctuidae) in Australia. J Econ Entomol. 2007; 100:894–902. 17598553 10.1603/0022-0493(2007)100[894:rtbttc]2.0.co;2

[12] Heckel DG. Learning the ABCs of Bt: ABC transporters and insect resistance to *Bacillus thuringiensis* provide clues to a crucial step in toxin mode of action. Pestic Biochem Phys. 2012; 104:103–110.

[13] Park Y, Gonzalez-Martinez RM, Navarro-Cerrillo G, Chakroun M, Kim Y, Ziarsolo P, et al ABCC transporters mediate insect resistance to multiple Bt toxins revealed by bulk segregant analysis. BMC Biol. 2014; 12:46 10.1186/1741-7007-12-46 24912445 PMC4071345

[14] Xiao Y, Zhang T, Liu C, Heckel DG, Li X, Tabashnik BE, et al Mis-splicing of the ABCC2 gene linked with Bt toxin resistance in *Helicoverpa armigera* . Sci Rep. 2014; 4:6184 10.1038/srep06184 25154974 PMC4143771

[15] Coates BS, Siegfried. Linkage of an ABCC transporter to a single QTL that controls *Ostrinia nubilalis* larval resistance to the *Bacillus thuringiensis* Cry1Fa toxin. Insect Biochem Mol Biol. 2015; 63: 86–96. 10.1016/j.ibmb.2015.06.003 26093031

[16] Gahan LJ, Gould F, Heckel DG. Identification of a gene associated with Bt resistance in *Heliothis virescens* . Science 2001; 293: 857–860. 11486086 10.1126/science.1060949

[17] Zhang H, Tian W, Zhao J, Jin L, Yang J, Liu C, et al Diverse genetic basis of field-evolved resistance to Bt cotton in cotton bollworm from China. Proc Natl Acad Sci USA. 2012; 109:10275–10280. 10.1073/pnas.1200156109 22689968 PMC3387040

[18] Fabrick JA, Ponnuraj J, Singh A, Tanwar RK, Unnithan GC, Yelich AJ, et al Alternative splicing and highly variable cadherin transcripts associated with field-evolved resistance of pink bollworm to Bt cotton in India. PLoS One. 2014; 9(5):e97900 10.1371/journal.pone.0097900 24840729 PMC4026531

[19] Tabashnik BE, Huang F, Ghimire MN, Leonard BR, Siegfried BD, Rangasamy M, et al Efficacy of genetically modified Bt toxins against insects with different genetic mechanisms of resistance. Nat Biotechnol. 2011; 29: 1128–1131. 10.1038/nbt.1988 21983521

[20] Monnerat R, Martins E, Macedo C, Queiroz P, Praça L, Soares CM, et al Evidence of field-evolved resistance of *Spodoptera frugiperda* to Bt corn expressing Cry1F in Brazil that is still sensitive to modified Bt toxins. PLoS ONE. 2015; 10(4): e0119544 10.1371/journal.pone.0119544 25830928 PMC4382162

[21] Downes S. 2014–15 end of season resistance monitoring report Australian Government Cotton Research and Development Corporation, 2015.

[22] Mahon RJ, Olsen KM. Limited survival of a Cry2Ab-resistant strain of *Helicoverpa armigera* (Lepidoptera: Noctuidae) on Bollgard II. J Econ Entomol. 2009; 102:708–716. 19449653 10.1603/029.102.0232

[23] Mahon RJ, Young S. Selection experiments to assess fitness costs associated with Cry2Ab resistance in *Helicoverpa armigera* (Lepidoptera: Noctuidae). J Econ Entomol. 2010; 103:835–842. 20568630 10.1603/ec09330

[24] Bird LJ, Downes SJ. Toxicity and cross-resistance of insecticides to Cry2Ab-resistant and Cry2Ab-susceptible *Helicoverpa armigera* and *Helicoverpa punctigera* (Lepidoptera: Noctuidae). J Econ Entomol. 2014; 107:1923–1930. 10.1603/EC14230 26309283

[25] Caccia S, Hernandez-Rodriguez CS, Mahon RJ, Downes S, James W, Bautsoens N, et al Binding site alteration is responsible for field-isolated resistance to *Bacillus thuringiensis* Cry2A insecticidal proteins in two *Helicoverpa* species. PLoS ONE. 2010; (3):e9975. 20376312 10.1371/journal.pone.0009975 PMC2848615

[26] Atsumi S, Miyamoto K, Yamamoto K, Narukawa J, Kawai S, Sezutsu H, et al Single amino acid mutation in an ATP-binding cassette transporter gene causes resistance to Bt toxin Cry1Ab in the silkworm, *Bombyx mori* . Proc Natl Acad Sci USA. 2012; 109: E1591–E1598. 10.1073/pnas.1120698109 22635270 PMC3382473

[27] Ohja A, Sowjanya Sree K, Sachdev B, Rashmi MA, Ravi KC, Suresh PJ, et al Analysis of resistance to Cry1Ac in field-collected pink bollworm, (Lepidoptera: Gelechiidae), populations. GM Crops and Food 2014; 5:4, 280–286. 10.4161/21645698.2014.947800 PMC5033187 25523173

[28] Kurmanath KV, Wily Pink Bollworm Survives Monsanto’s Bollgard-II. The Hindu Business Line. 28 October 2015. http://www.thehindubusinessline.com/economy/agri-business/wily-pink-bollworm-survives-monsantos-bollgardii/article7814810.ece. Accessed 31 October 2015.

[29] Tabashnik BE, D. Mota-Sanchez D, M. E. Whalon ME, R. M. Hollingworth RM and Y. Carrière Y. Defining terms for proactive management of resistance to Bt crops and pesticides. J Econ Entomol. 2014; 107: 496–507. 24772527 10.1603/ec13458

[30] Gassmann AJ, et al (2014) Field-evolved resistance by western corn rootworm to multiple *Bacillus thuringiensis* toxins in transgenic maize. Proc Natl Acad Sci USA 2014; 111:5141–5146. 10.1073/pnas.1317179111 24639498 PMC3986160

[31] Huang F, Qureshi JA, Meagher RL Jr., Reisig D, Head GP, Andow DA, et al Cry1F resistance in fall armyworm *Spodoptera frugiperda*: single gene versus pyramided Bt maize. PLoS ONE 2014; 9(11): e112958 10.1371/journal.pone.0112958 25401494 PMC4234506

